# Revisiting outcomes after transcatheter aortic valve replacement: The need for a multidimensional approach to patient management

**DOI:** 10.1016/j.ijcha.2026.101927

**Published:** 2026-04-22

**Authors:** Carmelita Cieri, Michele Russo, Marco Zimarino

**Affiliations:** a Department of Cardiology, SS. Annunziata Hospital, ASL2 Abruzzo, Chieti, Italy; b Department of Neuroscience, Imaging and Clinical Sciences, “Gabriele D’Annunzio” University of Chieti-Pescara, Chieti, Italy

**Keywords:** Transcatheter aortic valve replacement, Outcomes, Follow up, Comorbidities, Frailty

**Figure:**
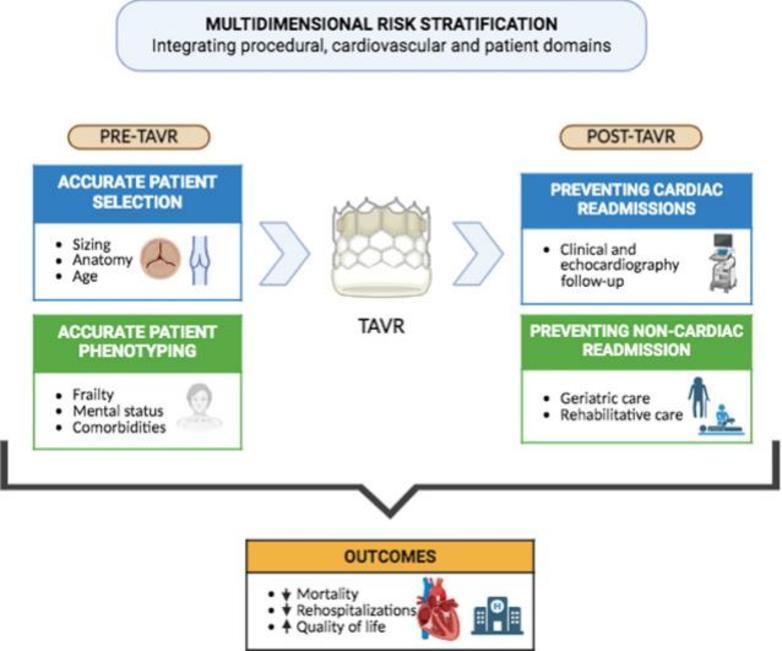

To Editor,

Transcatheter aortic valve replacement (TAVR) has become a widely performed and effective therapy for severe aortic stenosis, with continuous improvements in procedural safety and expanding use in older and more comorbid patients [1]. This shift has effectively moved the clinical frontier from procedural success to the challenges of long-term survivorship. However, despite these advances, the post-procedural course remains complex, and hospital readmissions continue to represent a substantial clinical burden. It has been estimated that about 44% of patients have an unplanned rehospitalisation over 1 year, with an incidence rate three-fold higher in the first 30 days after TAVR [2]. Importantly, both cardiovascular and non-cardiovascular causes contribute significantly to rehospitalisations, with the latter representing a major and often underrecognized contributor to post-TAVR risk. These events reflect not only procedure-related complications but also the impact of comorbidities, reduced physiological reserve, and overall patient vulnerability [3].

In this issue of *Int J Cardiol Heart & Vasculature*, Naito et al. [4] provide clinically relevant insights into the prognostic impact of cause-specific readmissions following TAVR. In a retrospective single-center cohort of 995 patients with long-term follow-up (up to 10 years), both cardiac (heart failure-related) and non-cardiac (including infections, fractures, and stroke) rehospitalisations were independently associated with increased mortality. Notably, non-cardiac events – particularly infections and fractures – showed a prognostic impact comparable to that of heart failure. These findings illuminate the 'competing risks' phenomenon: once the mechanical obstruction is relieved, the patient’s underlying vulnerability to non-cardiac insults becomes the primary determinant of prognosis. The identification of clinical factors associated with distinct patterns of rehospitalisation further refines our understanding of post-TAVR risk and suggests divergent pathways leading to cardiac and non-cardiac complications. These findings represent an important step toward more tailored post-procedural management.

The study also underscores the central role of patient vulnerability in shaping outcomes after TAVR. Frailty, a key component of this vulnerability, is highly prevalent in this population and is frequently accompanied by reduced physical performance, sarcopenia, and nutritional impairment, all of which are independently associated with mortality and impaired quality of life [5], [6]. Comorbid conditions – including chronic kidney disease, chronic obstructive pulmonary disease, atrial fibrillation, cardiac amyloidosis, and cognitive impairment – further contribute to adverse outcomes [4], [7]. These observations challenge a purely procedure-centered view and support a broader, patient-centered framework for risk assessment. Accordingly, pre-procedural evaluation should extend beyond anatomical and hemodynamic parameters to incorporate systemic and geriatric domains. This holistic perspective transitions the field from a valve-centric model to a comprehensive frailty-informed strategy (Fig. 1).

**Fig. 1:**
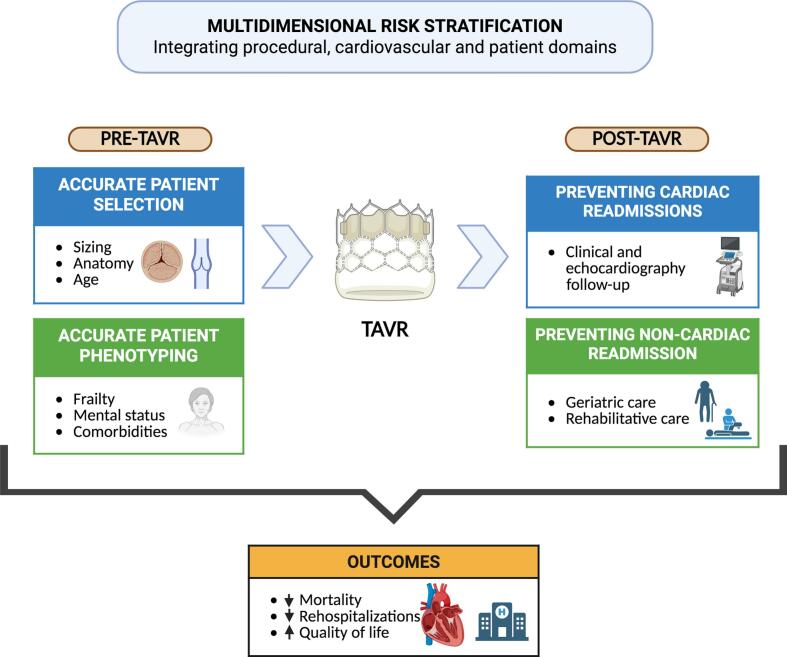
Xxxx.

While several risk models have been developed, however their predictive performance remains moderate, and most are primarily focused on mortality, with limited consideration of other clinically meaningful outcomes such as rehospitalisations. This limitation highlights the need for more comprehensive and multidimensional stratification strategies that better reflect the overall risk profile of TAVR patients [8]. In this context, even events traditionally considered minor may carry significant clinical implications, starting from the immediate periprocedural management, where unnecessary blood transfusions – often viewed as minor periprocedural incidents—actually represent significant subclinical insult – are associated with adverse short-term outcomes and may predispose patients to subsequent rehospitalisations [9].

Taken together, these findings call for a shift in clinical perspective. Procedural success alone is no longer sufficient. Durable benefit after TAVR requires refined patient selection and structured, longitudinal care. Advanced phenotyping – including frailty and comorbidity profiling – should be integrated with targeted preventive strategies addressing infections, falls, and other drivers of vulnerability. In this regard, optimizing antithrombotic regimens is paramount, as the use of single as compared to double antiplatelet therapy improves long-term survival beyond the mere reduction of bleeding events, suggesting that a simplified antithrombotic regimen may better preserve the fragile equilibrium of TAVR recipients [10]. In parallel, emerging tools such as tele-monitoring and digital health, potentially enhanced by artificial intelligence, may enable earlier detection of clinical deterioration and more personalized long-term management.

In this evolving landscape, the success of TAVR should be defined not only by procedural outcomes, but by its ability to deliver sustained, patient-centered benefit over time. Ultimately, the measure of TAVR’s success must evolve from simply adding years to life, to ensuring those years are defined by functional independence and quality of survival.

## CRediT authorship contribution statement

**Carmelita Cieri:** Conceptualization, Writing – original draft. **Michele Russo:** Writing – original draft, Writing – review & editing. **Marco Zimarino:** Supervision, Validation.

## Funding

None.

## Declaration of competing interest

The authors declare that they have no known competing financial interests or personal relationships that could have appeared to influence the work reported in this paper.
